# Brain Activation of Elite Race Walkers in Action Observation, Motor Imagery, and Motor Execution Tasks: A Pilot Study

**DOI:** 10.3389/fnhum.2019.00080

**Published:** 2019-03-01

**Authors:** Qihan Zhang, Peng Zhang, Lu Song, Yu Yang, Sheng Yuan, Yixin Chen, Shinan Sun, Xuejun Bai

**Affiliations:** ^1^Academy of Psychology and Behavior, Tianjin Normal University, Tianjin, China; ^2^College of Educational Science, Tianjin Normal University, Tianjin, China; ^3^Center of Collaborative Innovation for Assessment and Promotion of Mental Health, Tianjin, China

**Keywords:** action observation, motor imagery, motor execution, race walking, functional near-infrared spectroscopy (fNIRS)

## Abstract

Walking plays an important role in human daily life. Many previous studies suggested that long-term walking training can modulate brain functions. However, due to the use of measuring techniques such as fMRI and PET, which are highly motion-sensitive, it is difficult to record individual brain activities during the movement. This pilot study used functional near-infrared spectroscopy (fNIRS) to measure the hemodynamic responses in the frontal-parietal cortex of four elite race walkers (experimental group, EG) and twenty college students (control group, CG) during tasks involving action observation, motor imagery, and motor execution. The results showed that activation levels of the pars triangularis of the inferior frontal gyrus (IFG), dorsolateral prefrontal cortex (DLPFC), premotor and supplementary motor cortex (PMC and SMC), and primary somatosensory cortex (S1) in the EG were significantly lower than in the CG during motor execution and observation tasks. And primary motor cortex (M1) of EG in motor execution task was significantly lower than its in CG. During the motor imagery task, activation intensities of the DLPFC, PMC and SMC, and M1 in the EG were significantly higher than in the CG. These findings suggested that the results of motor execution and observation tasks might support the brain efficiency hypothesis, and the related brain regions strengthened the efficiency of neural function, but the results in motor imagery tasks could be attributed to the internal forward model of elite race walkers, which showed a trend opposed to the brain efficiency hypothesis. Additionally, the activation intensities of the pars triangularis and PMC and SMC decreased with the passage of time in the motor execution and imagery tasks, whereas during the action observation task, no significant differences in these regions were found. This reflected differences of the internal processing among the tasks.

## Introduction

Walking is the most repeated and complex holistic movement in human daily activity. It reflects the coordination of an individual’s motor-related brain structure and function and also plays an important role in the quality of life and physical and mental health of the individual (Whittle, 1993; Lee and Buchner, 2008; Axer et al., 2010).

Race walking is a sport modeled after the movement of regular walking. Its rules require (1) the supporting leg to be straightened upon first contact with the ground until the body passes over the foot and (2) the back toe not to leave the ground until the heel of front foot has contacted the ground, ensuring that both feet are not simultaneously off the ground. The rule of a straightened supporting leg differentiates race walking from walking.

Studies on elite race walkers have mainly focused on the detection of speed, duration, ground reaction, joint power, heart rate, oxygen uptake, and other kinematic indicators or peripheral nerve activity (Hanley et al., 2013; Pavei et al., 2014; Gomez-Ezeiza et al., 2018). Evaluations of brain activity during race walking and the differences between elite race walkers and ordinary people are rare. Persistent training of such elite athletes not only promotes the formation of new movement patterns and changes in the speed–accuracy relationship (Willingham, 1998; Reis et al., 2009; Chein and Schneider, 2012; Shmuelof et al., 2012; Telgen et al., 2014; Diedrichsen and Kornysheva, 2015) but also leads to changes in brain structure and function (Del Percio et al., 2008; Yarrow et al., 2009; Nakata et al., 2010; Callan and Naito, 2014).

To study the neurological activity of individuals with high-level motor skills, previous studies often used classic expert-novice paradigms. Highly motion-sensitive measuring techniques such as electroencephalography (EEG), functional magnetic resonance imaging (fMRI), or positron emission tomography (PET) were employed to monitor and compare the neural activation of individuals with high-level motor skills or low-level motor skills at a resting state or while completing a simple task. For example, the brain activation patterns in rifle shooters (Di Russo et al., 2005), gymnasts (Huang et al., 2018), or archers (Chang et al., 2011) compared with ordinary people were assessed based on two types of experimental tasks: (1) resting-state tasks unrelated to motor cognition that determined the extent to which exercise training can affect an individual’s brain function and (2) tasks related to motor cognition, including motor execution, motor imagery, and action observation. PET, EEG, and fMRI measurements are extremely sensitive to an individual’s movement, which adds challenges to studying brain activity during exercise. To circumvent these movement-associated challenges, Di Russo et al. (2005) conducted a study on elite shooters and non-athletes, monitoring their neural activity while they completed micro-motor tasks (e.g., self-paced flexion movements). In studies of lower-limb movements, brain activity was assessed while the participants completed simple tasks such as toe or foot swinging and foot-pressing on objects (Orr et al., 2008; Hotz-Boendermaker et al., 2011). However, these tasks differ greatly from the limb movements used in daily life.

To overcome the impact of movement on the acquisition of signals, researchers have used motor imagery or action observation tasks (that do not require physical movement by the test participants) instead of motor execution tasks to investigate the differences in brain activity between individuals with high- and low-level motor skills. For example, Kraeutner et al. (2018) recruited basketball players, volleyball players, and ordinary individuals to complete motor imagery tasks. In another study, Olsson and Lundström (2013) required hockey players and non-hockey playing controls to complete action observation tasks.

It is not known whether the results of motor imagery and action observation tasks represent the brain activation patterns of individuals during exercise. Jeannerod (2001) proposed the mental simulation theory that suggested a functional equivalence between motor execution, motor imagery, and action observation. Since simulation of body movement is based on motor representations generated in an individual’s brain, the mental simulation theory postulates that functional equivalence is present between the three tasks: motor imagery, action observation, and motor execution.

Studies have shown that the neural representation of motor imagery and action observation is similar to that of motor execution (Filimon et al., 2007; Szameitat et al., 2007; Gazzola and Keysers, 2009; Jarstorff et al., 2010). However, a meta-analysis by Hardwick et al. (2017) showed that motor imagery, action observation, and motor execution tasks all activate the premotor–parietal network and the somatosensory network, but their activation of the motor cortex, parietal cortex, and subcortical structures vary significantly. These activation differences may correspond to a disparity in the specific focus of intrinsic processing between action observation, motor imagery, and action execution. Action observation serves to understand the movements and intentions of others through the brain’s mirror neuron system (MNS), thereby causing an individual to imitate the movements (Fabbri-Destro and Rizzolatti, 2008; Rizzolatti and Fabbri-Destro, 2008; Caspers et al., 2010). Motor imagery is a psychological simulation of movements, emphasizing the preparation and planning before the movement output and suppression of execution the motion commands (Hétu et al., 2013). Motor execution is the output of movements co-regulated by bottom-up sensory feedback and top-down subjective intention (Scott, 2016). Collectively, there are partial overlaps of the neural mechanisms of action observation, motor imagery, and motor execution, but the differences among these tasks might be caused by their differences in internal processing.

With the continuous development of brain imaging techniques, researchers have used functional near-infrared spectroscopy (fNIRS) to monitor the hemodynamic responses in the cerebral cortex of athletes. Its greatest advantage is that it can investigate cortical responses during exercise (Leff et al., 2011) or other types of holistic movements. Elite race walkers already have learnt very good forward models, i.e., the capability to perform good representation, preparation, planning, and monitoring of upcoming movements in any given context (Yarrow et al., 2009). Hence, the experimental tasks we chose for the current study included a motor execution task (actual race walking) that simulated scenarios from the actual sport, as well as action observation and motor imagery tasks that represented internal cognitive processes such as movement understanding, motor preparation, and motor planning. In order to determine the specific brain activity of elite race walkers, this pilot study compared the differences in cerebral activation between these top athletes and college students completing action observation, motor imagery, and motor execution tasks.

The brain efficiency hypothesis proposes that participants with high ability show the largest decreases in brain glucose metabolic rates (Haier et al., 1992). In other words, the reduction in neural activity caused by long-term physical training reflects an increase in the efficiency of neural function (Poldrack, 2015). This hypothesis has been confirmed in elite athletes in a motor cognitive task. For example, Di Russo et al. (2005) found that the amplitudes of motor-related cortical potentials in elite shooters were smaller than in non-athletes during movements of the right fingers. Guo et al. (2017) examined table tennis athletes and non-athletes performing visuo-spatial tasks with fMRI. The results of neuroimaging supported the brain efficiency hypothesis. Since elite race walkers have completed many years of rigorous training, the motor-related tasks in this pilot study were basically a repetition of their training exercises. Hence, we hypothesized that the elite race walkers would likely show a decrease in related cortical activation during the motor-related tasks compared to the control group (CG).

## Materials and Methods

### Participants

In this pilot study, the athletes were selected according to their sport ranks and titles in order to restrict the experimental group (EG) to elite race walkers with International Master of Sports or Master of Sports titles. Non-physical education students who had no rigorous or regular exercise training were recruited for the CG. All the pilot study participants were male, with a visual acuity or corrected acuity above 1.0 and no color blindness. None of the participants had a previous history of cardiovascular, pulmonary, renal, neurological, psychiatric, or other severe diseases that might otherwise have influenced the experimental results, and did not consume caffeinated food or drink before the start of the experiment. The Movement Imagery Questionnaire and the Edinburgh Handedness Inventory (Oldfield, 1971) were used as screening tools to ensure that only right-handed individuals with a mid-level of motor imagination (or higher) were enlisted; specifically, this pilot study included only those participants who received scores > 5 (“somewhat easy”) in the MIQ and scores > 40 (right-handedness) in the Edinburgh Handedness Inventory. The final four elite race walkers recruited in the EG were an Olympic champion and runner-up in the Men’s 20 km race walk, and the winners of the National 50 and 20 km race walks. All the athletes were Chinese men with an average age of 23.75 years and an average imagination score of 5.75. The CG was comprised of twenty non-physical education students from a college in Tianjin, China, with an average age of 22.65 years and an average imagination score of 5.86. All participants in the pilot study were approved by the Ethics Committee of Tianjin Normal University, and the pilot study was performed strictly in accordance with the approved guidelines. The subjects provided written informed consent prior to starting the experiments and obtained a cash reward after the experiments.

### Experimental Equipment

An fNIRS system manufactured by Shimadzu Corporation (LABNIRS/16, Shimadzu Corporation, Kyoto, Japan) was used in this study. A 3-wavelength (780 ± 5 nm, 805 ± 5 nm, and 830 ± 5 nm) semiconductor laser system (1M level under the IED-60825-1 standard) was used to monitor changes in cortical hemoglobin concentrations according to the modified Beer-Lambert Law (MBLL). The hemoglobin concentrations were measured based on three indicators: oxy-hemoglobin (HbO), deoxy-hemoglobin (HbR), and total hemoglobin (HbT). The sampling rate of this pilot study was 11 Hz. Hoshi et al. (2001) showed that HbO is more sensitive to changes in brain activity during task simulation than HbR and HbT; therefore, we chose to analyze the changes in HbO concentration in the cortex under our various experimental conditions.

The MIQ was composed of seven kinesthetic imagery questions in the modified *MIQ-RS* (Gregg et al., 2010), scored on a 7-point scale from 1 (“very difficult”) to 7 (“very easy”). The Self-edited Movement Imagery Self-assessment Questionnaire was composed of four questions that were used to assess the imaginative capabilities of the participants while they completed the motor imagery task. Participants were asked to comment on the degree of difficulty, vividness, attention, and physical exertion using a ranking from 1 (“very difficult/not vivid/not focused/less physical exertion”) to 7 (“Very easy/vivid/focused/great physical exertion).

The race-walking video used in the action observation task was a 2 min excerpt from the International Association of Athletics Federations (IAAF) World Race Walking Team Championships. The participants in the EG did not appear in this video. To prevent any auditory stimulation from interfering with the cortical activity of the participants, the race-walking video was played on mute.

### Experimental Procedures

This study adopted a 2 (subject groups, i.e., EG and CG) × 3 (motor tasks, i.e., motor execution, motor imagery, and action observation) mixed design.

Prior to the formal experiment, the participants were asked to participate in a practice session by race walking on a treadmill at a self-controlled pace (minimum speed of 4 km/h) for 1 min, followed by a rest period. After this rest, the participants were asked to imagine for 1 min the physical feeling they experienced during the previous race walking. The cycle of “motor execution–rest–motor imagery–rest” was repeated many times until the participants were able to complete the motor imagery task. During the cycles of motor imagery, the interviewer occasionally asked the participants about their current physical feeling (e.g., is your current speed fast or slow?) for evaluating the quality of the motor imagery.

**FIGURE 1:**
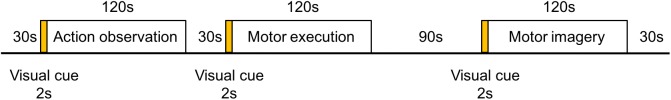
Flow chart of the experimental paradigm. The experimental paradigm was divided into three tasks of motor execution, action observation, and motor imagery. The sequence of the tasks was randomized. A visual cue for 2 s was presented in the middle of the monitor as a signal to progress to the next task.

Following the practice session, the participants rested for 5 min, and then they performed the formal experiments. In the formal experiment, the participants were asked to rest for 30 s and were then shown a visual cue on a screen for 2 s representing the upcoming task (motor execution, action observation, or motor imagery). After the visual cue disappeared, the participants were required to execute the cued task for 120 s. The action observation task was watching the video of race walking, the motor execution task was race walking on a treadmill at a self-adjusted pace (at least 4 km/h), and the motor imagery task was to imagine this self-paced race walk. For safety reasons during the motor execution task, participants were not allowed to stop too abruptly on the treadmill and a buffer time was provided for them to slow to a stop. Accordingly, the rest period following the motor execution task was extended to 90 s (Figure 1). During the action observation task and motor imagery task, the hands of the participants were placed on (but not grasping) the two buttons from the left and right sides of the treadmill, their feet were close together, and their bodies were upright. A vertical plane containing the two buttons overlapped the coronal plane of the participants’ bodies, and their feet were placed on the middle line between the two buttons. The participants were instructed not to move their body or head during the action observation task, motor imagery task, or rest period. In addition, the sequence of the three tasks was randomized for the formal experiments. After completing the experiment, each participant was asked to fill out the Movement Imagery Self-assessment Questionnaire regarding his performance in the motor imagery task.

### Probe Arrangement

**FIGURE 2:**
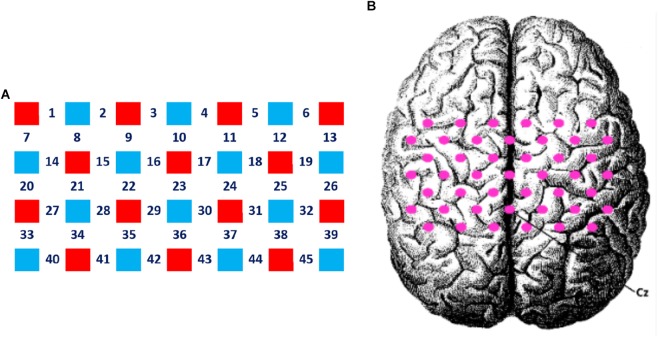
Layout of the fNIRS channels. **(A)** The red block represents the emitter and the blue block represents the detector. Twenty eight optodes (14 emitters and 14 detectors) were attached to the frontal-parietal cortex in a 4 × 7 multi-channel probe holder forming 45 measurement channels. **(B)** The pink dot represents the measurement channels, following the international 10–20 system. Channel 36 was placed at the Cz position.

A 4 × 7 multi-channel probe holder containing 14 emitters and 14 detectors was used in this study, with a 3 cm distance between probes, forming a total of 45 channels covering the frontal-parietal cortex. Following the international 10–20 system, channel 36 was placed at the Cz position (Figure 2, the red block represents the emitter and the blue block represents the detector). The three-dimensional FASTRAK Locator (Polhemus FASTRAK, Colchester, VT) was used to determine the coordinate points (Cz, Nz, AL, and AR) and probe locations. Each channel position was registered with the Montreal Neurological Institute (MNI) coordinates using the probability registration method in order to determine the corresponding Brodmann area (BA).

### Data Analysis

To eliminate the sporadic interference factors affecting the signal at the beginning and the end of each task, fNIRS signals in the first and the last 10 s of the action observation, execution, and imagery tasks were not analyzed. Specifically, only 100 s of fNIRS data in the middle of the task performance was processed and analyzed. It is well known that a relationship exists between regional cerebral blood flow (CBF, i.e., to measure the arterial diameter) and neural activity, with arterial diameter tending to rise initially and then decrease, while neural activity continues (Ngai et al., 1988). Therefore, in order to investigate the time variations of each channel during different tasks, we divided the 100 s fNIRS data stream into two parts: (1) the first 30 s of signal and (2) the last 70 s of signal. In addition, since the changes in the blood-oxygen signal were slow, it took some time to fall back to the baseline level. To ensure the stability of the baseline, the last 5 s of the rest period was selected as the baseline signal of the cortical blood-oxygen concentration in each individual.

First, the extracted fNIRS signal was denoised and drifted using the Wavelet-minimum description length method (Jang et al., 2009). Specifically, the NIRS-Statistical Parametric Mapping (NIRS-SPM) toolbox (version; v.4.1) was used in the Matlab (v.2012b) operating environment (Ye et al., 2009). The drift and noise (e.g., head movements and heart rate) were eliminated by wavelet analysis and Hemodynamic Response Functions (HRF), followed by setting the reference wave. The degree of the reaction induced by the experimental tasks in response to the reference wave (beta value) on each channel was evaluated by the General Linear Model (GLM), and the temporal autocorrelation of this process was adjusted using the Pre-coloring method.

Second, according to the experimental design, R (v.3.3.2) and RStudio (v.1.0.136) were used to perform repeated-measures analysis of variance (ANOVA) tests, permutation tests, exact Wilcoxon signed rank tests, and one-way permutation test based on 9999 Monte-Carlo resamplings in the obtained beta values for the different subject groups and different motor tasks. Given the small sample size of the EG (*n* = 4) and CG (*n* = 20) with unknown overall distributions, a permutation test was used to recalculate the statistical quality, construct the empirical distribution, and determine the *P*-value (Wheeler and Torchiano, 2016). The *P*-value was additionally subjected to a false discovery rate (FDR) correction. The above calculations were mainly carried out by the lmPerm and the coin packages (Hothorn et al., 2008; Wheeler and Torchiano, 2016).

Finally, the results of the experiments were visualized using the “BrainNetViewer” tool in Matlab (v.2012b) and the “sciplot” software package in Rstudio (v.1.0.136) (Xia et al., 2013; Morales, 2017).

## Results

### Analysis of Questionnaire Data for the Motor Imagery Task

The exact single-factor permutation test was used to analyze the data from the MIQ and the Movement Imagery Self-assessment Questionnaire in the EG and the CG. Our results showed no significant differences of kinesthetic imagination (*z* = 0.271), difficulty (*z* = 1.751), vividness (*z* = −0.363), attention (*z* = −0.560), or degree of physical exertion (*z* = 0.271) in the motor imagery task between the EG and CG (*P* > 0.05). The mean questionnaire score was 5.750 ± 0.393 and 5.857 ± 0.778 for the EG and CG, respectively.

### Data Analysis of the fNIRS Signals

We conducted a permutation test for repeated-measures ANOVA and FDR correction of the beta values evaluated by the GLM model adopting 2 subject groups (i.e., EG and CG) × 3 motor tasks (i.e., motor execution, motor imagery, and action observation) × 2 time (i.e., 30 and 70 s). The results of the analysis are shown in Table 1, where the *P*-values listed were calculated after FDR correction. The following results were observed:

**Table 1:** The interaction results of each channel under different experimental conditions.

Channel	MNI			L/R	BA	Overlapped	*F*	*p* (corrected)
	*X*	*Y*	*Z*					
**Interaction: Motor Tasks × Time**								
6	48	43	28	R	45	0.770	8.171	0.000
7	−51	34	27	L	45	0.989	12.179	0.000
13	55	31	28	R	45	0.878	19.866	0.000
20	−56	9	41	L	6	0.652	6.547	0.047
26	60	4	42	R	6	0.803	8.149	0.002
**Interaction: Motor Tasks × Subject Groups**								
1	−43	46	29	L	46	0.502	7.082	0.021
2	−26	46	43	L	9	0.927	8.836	0.014
3	−9	48	51	L	9	0.865	6.514	0.022
4	13	48	51	R	9	0.851	5.520	0.029
5	32	45	44	R	9	0.940	10.531	0.008
6	48	43	28	R	45	0.770	7.886	0.024
7	−51	34	27	L	45	0.989	8.090	0.019
9	−17	36	57	L	8	0.733	8.411	0.019
11	23	36	57	R	8	0.722	5.860	0.029
12	42	32	46	R	9	0.841	8.908	0.008
13	55	31	28	R	45	0.878	17.981	0.008
14	−47	24	46	L	9	0.599	5.030	0.031
17	14	26	65	R	8	0.849	5.330	0.029
18	34	23	59	R	8	0.660	6.944	0.019
19	49	21	47	R	9	0.706	8.264	0.014
20	−56	9	41	L	6	0.652	8.082	0.019
21	−42	13	59	L	6	0.466	7.611	0.020
23	2	12	69	R	6	0.976	6.516	0.021
25	44	11	59	R	6	0.466	20.865	0.000
26	60	4	42	R	6	0.803	10.261	0.007
27	−51	−2	55	L	6	0.932	5.901	0.028
29	−13	−1	75	L	6	1.000	9.204	0.005
30	15	0	75	R	6	1.000	7.785	0.011
32	54	−6	56	R	6	0.647	6.463	0.023
33	−61	−19	48	L	1,2,3	0.829	5.219	0.031
34	−44	−18	66	L	4	0.643	4.511	0.049
36	0	−16	74	L	6	0.707	14.054	0.007
37	24	−16	76	R	6	0.909	6.355	0.029
38	46	−18	66	R	4	0.645	33.830	0.000
39	64	−23	49	R	1,2,3	0.825	9.430	0.014
41	−35	−29	72	L	4	0.683	10.535	0.007
42	−13	−32	80	L	4	0.852	5.075	0.038
43	14	−32	80	R	4	0.844	17.016	0.000
44	35	−30	73	R	4	0.706	12.857	0.008
45	55	−32	58	R	1,2,3	0.675	14.646	0.006

**FIGURE 3:**
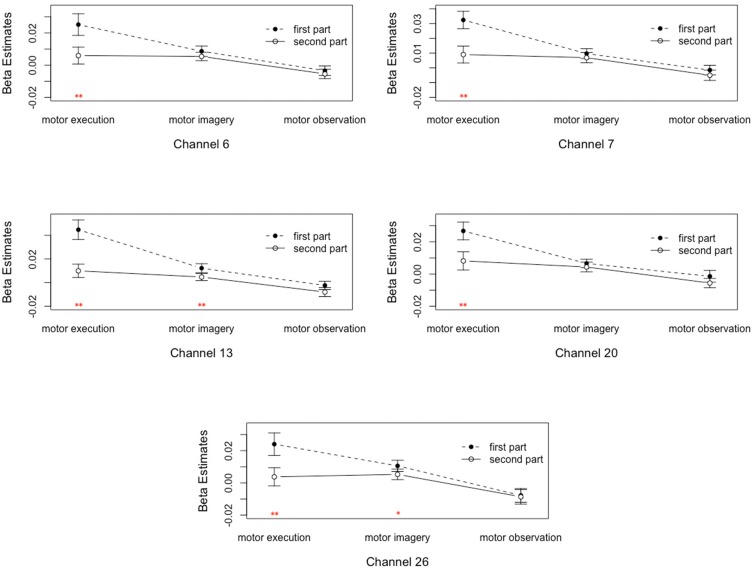
Analysis of the interactions between the motor tasks and time. Error bars represent standard errors. ^∗^*P* < 0.05, ^∗∗^*P* < 0.01.
(4) The main effect of subject groups was significant; the activation intensities of the pars triangularis, DLPFC, PMC and SMC, S1 and M1 of the EG were significantly lower than those of the CG (details see Supplementary Material).
(5) Table 1 and Figure 4 shows the channels with a significant interaction between the motor tasks and subjects. The one-way permutation test of the different motor tasks in the EG and CG showed that activation levels of the pars triangularis, DLPFC, PMC and SMC, S1, and M1 (channel of 2, 3, 4, 5, 6, 9, 13, 17, 20, 23, 26, 29, 30, 32, 36, 38, 39, 42, and 44) of the EG were significantly lower than the activation of those same channels of the CG in the motor execution task. In the motor imagery task, the activation of DLPFC, PMC and SMC, and M1 (channel of 1, 2, 3, 4, 5, 9, 11, 12, 17, 18, 21, 27, 29, 30, 34, and 41) of the EG was significantly higher than that of the CG. In the action observation task, the activation intensity of channels associated with subjects, including pars triangularis, DLPFC, PMC and SMC, and S1 (channel of 1, 2, 3, 4, 5, 6, 9, 11, 13, 14, 17, 19, 20, 23, 26, 29, 30, 32, 33, 36, and 39) of the EG was significantly lower than that of the CG (details in Figure 5).

(1) The main effect of motor tasks was significant on the pars triangularis of the inferior frontal gyrus (IFG), dorsolateral prefrontal cortex (DLPFC), premotor and supplementary motor cortex (PMC and SMC), primary somatosensory cortex (S1), and primary motor cortex (M1). Its reflected the differences among these tasks are caused by their internal processing. For details, see Supplementary Material.
(2) The main effect of time was significant on channel 7 and 20, corresponding to the pars triangularis and PMC and SMC. This suggested that the signal intensities of channel 7 and 20 in the first 30 s were significantly higher than the last 70 s.
(3) The interaction between motor tasks and time was mainly reflected in the pars triangularis (Channel 6, 7, 13) and PMC and SMC (Channel 20, 26), see in Table 1. On the basis of the channels, the exact Wilcoxon signed rank test performed on the first 30 s and the last 70 s of data showed that the signals on these channels in the first 30 s of the motor execution were significantly higher than the signals of the channels in the last 70 s. And there were significant differences among the signals on channel 13 and 26 of the motor imagery. In contrast, no significant differences in the channels were found between the first 30 s and the last 70 s of data recorded during the action observation task (details in Figure 3).

**FIGURE 4:**
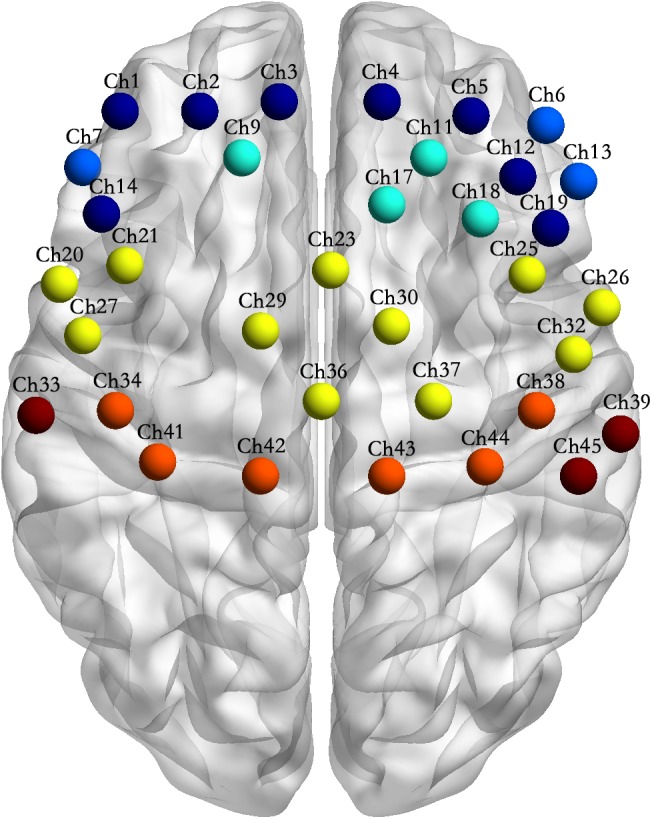
Channel location of the interactions between motor tasks and subject groups. Note: Channel sites of the same color in the figure represent that they are from the same Brodmann area (BA).

**FIGURE 5:**
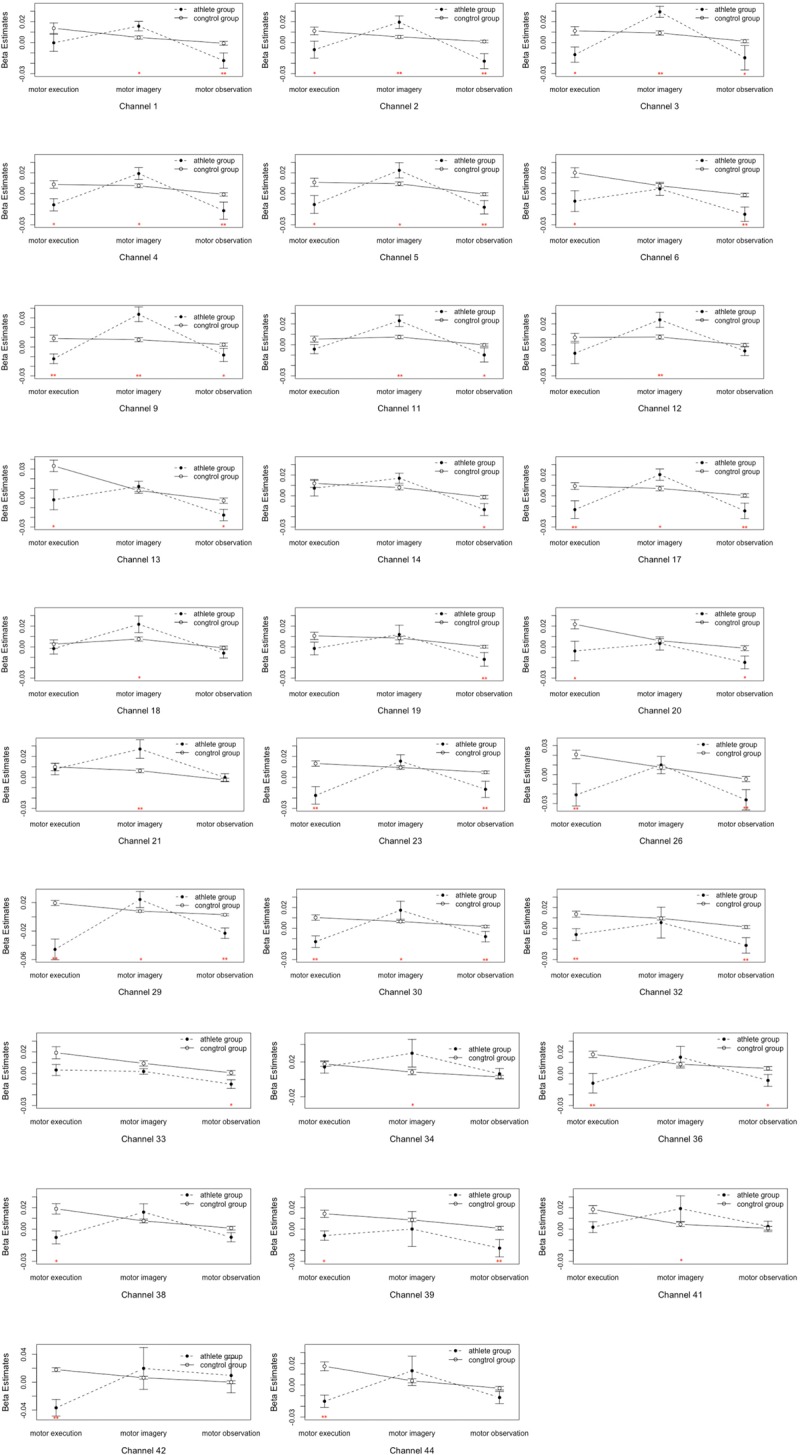
Analysis of the interactions between the motor tasks and subject groups. Error bars represent standard errors. ^∗^*P* < 0.05, ^∗∗^*P* < 0.01.

## Discussion

### Relationship Between the Subjects and Motor Tasks

The purpose of this pilot study was to investigate the brain activation in the EG and CG during some motor cognitive tasks. However, the presented data is only from a pilot study, a great caution should be undertaken when generalizing and interpreting the results.

Our hypothesis was based on the brain efficiency hypothesis. Specifically, we tested whether there was less cortical activity in the EG than in the CG during the motor execution, motor imagery, and action observation tasks. However, the results partly proved the hypothesis. Neuroimaging data demonstrated that the pars triangularis, DLPFC, PMC and SMC, S1, and M1 were regions with significant interactions between subjects and motor tasks. This comparison showed that the activation levels of the pars triangularis, DLPFC, PMC and SMC, and S1 of the EG were significantly lower than the CG in the motor execution and action observation tasks. And the activation levels of M1 in EG were significantly lower than the CG in the motor execution tasks. In the motor imagery tasks, the activation intensity of the DLPFC, PMC and SMC, and M1 of the EG were significantly higher than the CG. The findings suggested that the results of motor execution and observation tasks might have supported the brain efficiency hypothesis, which states that related brain regions strengthen the efficiency of neural function. However, the results in the motor imagery task showed a trend opposite to the brain efficiency hypothesis. This hypothesis also has been challenged by some other evidence. For example, Woods et al. (2014) examined the neural response to familiar sports and unfamiliar tasks in elite athletes and novice athletes, and found greater activation in elite athletes than in novice athletes during familiar tasks. Del Percio et al. (2008) used EEG to record the motor-related cortical potentials of fencers, karate athletes, and non-athletes. The results showed that amplitudes of the potentials overlying SMA and contralateral S1/M1 were lower in elite fencers and karate athletes than in non-athletes, but the amplitudes over the ipsilateral hemisphere were higher in the elite karate athletes than in the fencers and non-athletes. This difference might be related to practice-related decrease, increase, redistribution, and reorganization of brain activation of internal processes (Hardwick et al., 2013).

Jeannerod (2001) proposed the mental simulation theory that suggested a functional equivalence between motor execution, motor imagery, and action observation, and assumed that these are based on the similar action representation encoded. However, there are some differences in the mechanism of internal processes between them. Action observation is a percept-driven process to understand the movements and intentions of others (Caspers et al., 2010), which is guided by external stimuli. But motor imagery is guided by internal stimuli, relying on the individual’s long-term memory, and is part of a knowledge-driven process (Kim et al., 2017). Motor execution is the output of movements co-regulated by bottom-up sensory feedback (a percept-driven process) and top-down subjective intention (Scott, 2016). A more complete description of the differences is offered by the internal forward models, which are able to predict changes in the state of the body or objects around the body ahead of their action (Wolpert et al., 1995). In motor execution and action observation tasks, these predictions are effectively combined with the actual sensory feedback (e.g., sense of proprioception, visual, touch for motor execution, and sense of visual for action observation) to give a more accurate body or object state. Internal forward models can also predict the sensory outcome of an action without actually executing it. Grush (2004) proposed that motor imagery drives an emulator of body when the emulator receives an efference copy, it releases an output signal same as an actual sensory feedback signal. Elite race walkers generally have well-developed feedforward capabilities and is better at movement representation, preparation, and planning than a person in the CG (Yarrow et al., 2009). Therefore, we concluded that long-term training will promote individuals’ percept-driven and knowledge-driven processes. In percept-driven processes, an actual sensory stimulus (from the external environment) appeared repeatedly to lead neural pruning to improve the efficiency of neurons in related regions. This might be the reason why the related cortical areas were less active in the EG than in the CG during the motor execution and action observation tasks. However, the effects of percept-driven and knowledge-driven processes in long-term training are different. Along with the training, the levels of an individual’s knowledge with race walking was improved gradually, so the EG’s emulator can use much more information from their long-term memory than the CG to perform the motor imagery task precisely. Therefore, the EG needs a higher level of region-related activities for processing the information to complete the motor imagery task.

The prefrontal lobe is the core region for cognitive control processing and is capable of regulating individual behaviors from top to bottom (Miller and Cohen, 2001), involving, the pars triangularis, DLPFC, and other regions. Neuroimaging studies have provided evidence of activation in the pars triangularis when humans observe and execute actions (Dinstein et al., 2007). The pars triangularis of the IFG is not only the core brain region for the mirror system but also plays a significant role in the strategy integrating different movements (Dippel and Beste, 2015) and response suppression (Aron et al., 2004). The activation network pattern of action observation and motor imitation overlaps in the pars triangularis (Caspers et al., 2010), which also participate in the suppression of movement imitation by individuals during the action observation task. This pars triangularis is also involved in the motor execution task, since race walking is a complex movement of the whole body that requires integrated limb movement. The EG may have stronger efficiency of neural function in this area than the CG. Thus, the activation levels of the EG during the action observation and execution tasks were lower than that of the CG.

The DLPFC is primarily involved in the intrinsic processing of executive functions such as planning and working memory (Swick et al., 2011; Nejati et al., 2017) and is a core region of the cognitive control brain network (Chein and Schneider, 2012). The activation level of the DLPFC in movement learning follows an inverted U-shaped curve. In the initial stage of a movement, the participation level of the cognitive control network gradually increases over time. When the movement execution can be controlled, the participation level reaches its peak. Finally, as the repeated movement becomes increasingly automatic, activation of the cognitive control network gradually decreases (Chein and Schneider, 2012). This may be the reason why the activation intensity of the DLPFC in the EG was lower than that of the CG during the motor execution and action observation tasks. In contrast, motor imagery is a process of internal movement simulation without external action (Jeannerod, 1994, 2006). Large amounts of cognitive resources are needed to maintain and monitor the movement preparation and planning of an individual. Since the EG already have learnt very good internal forward models, their motor planning in motor imagery task may be more accurate than that of the CG, and more cognitive resources and monitoring were required to complete the motor imagery task, thereby showing higher activation levels in the EG than in the CG.

The motor-related brain regions observed in this study included the S1, M1, and PMC and SMC. The S1 plays a critical role in processing afferent somatosensory input and contributes to the integration of sensory and motor signals necessary for skilled movement (Borich et al., 2015). Our results showed that the activation level of the S1 of the EG during the motor execution and action observation tasks was significantly lower than in the CG. This might suggest that the neural function efficiency of the S1 responsible for somatosensory information processing was enhanced.

The M1 is a center of motor execution, and its major function is to pass motion instructions to effectors (Hanakawa et al., 2003). A previous study on the brain mechanisms of motor execution and motor imagery showed that the M1 was not only responsible for the execution of motion instructions but also participates in intrinsic processing such as motor planning and motor preparation (Hardwick et al., 2017). Thus, the M1 may play different roles during the tasks.

Human PMC and SMC refers to human BA6 and BA8 (Schubotz and Cramon, 2003). The PMC and SMC are involved in individual motor learning as well as cognitive control (Nachev et al., 2008). Many investigations of action observation have confirmed that these brain regions are the primary nodes of the MNS (Gallese et al., 1996; Molenberghs et al., 2009; Shaw et al., 2012; Balser et al., 2014; Hardwick et al., 2017). They are also activated during internal generation of motor timing (Macar et al., 1999). In addition, they can regulate the execution of motor strategies predicted by the forward model and are closely related to motor preparation, motor planning, and motor strategy conversion (Hoshi and Tanji, 2007; Yarrow et al., 2009; Roberts and Husain, 2015). Accordingly, these brain regions are critical in selecting appropriate motor responses and selectively suppressing the inappropriate motor responses (Mostofsky and Simmonds, 2008). They can also encode the speed and direction of executed movements (Tankus et al., 2009). Taken together, these findings suggest that the functions of the PMC and SMC involve the intrinsic processing of action observation, motor imagery, and motor execution.

### Relationship Between the Motor Tasks and Time

A previous study in an animal model showed that regional CBF and neural activities of the corresponding brain regions demonstrated an inverted U-shaped, asymmetrical relationship; for example, Ngai et al. (1988) observed the pial vascular response in the somatosensory cortex of the rat during stimulation of the sciatic nerve. And they found that, in the 60 s lower intensity (0.15 V) sustained stimulation period, the arterioles dilated abruptly after a 7 or 8 s delay to its peak response, subsequently decreased and maintained at a certain level, and then gradually returned to their baseline diameter when the external stimuli-evoked neural activity ended. To eliminate the sporadic interference factors that affect the beginning and the end of each task, fNIRS signals in the first and the last 10 s were eliminated, and the remaining signal (divided into the first 30 s and the last 70 s) was evaluated. The main effects of time and the interaction effects between time and motor tasks were found in the pars triangularis and PMC and SMC. Specifically, the activation intensities of the pars triangularis and PMC and SMC decreased with the passage of time in the motor execution and imagery tasks, whereas during the action observation task, no significant differences in the region were found. The pars triangularis and PMC and SMC were active during the motor execution and imagery tasks. This demonstrated regional CBF with the inverted U-shaped, asymmetrical changes accompanying sustained neural activity. However, in the action observation task, none of the detected brain regions showed the above changes over time. This shows that the changes in the related regions’ activity with time caused by the experimental tasks are different. This reflects the differences in internal processing between the tasks. The further reasons for this remain to be explored in future studies.

Taken together, the findings of this pilot study partly support the brain efficiency hypothesis. Specifically, the brain efficiency hypothesis is applicable for motor execution and action observation, which relate to the percept-driven processing, and is not enforceable in motor imagery within knowledge-driven processing. In addition, differences in internal processing during the tasks also lead to dissimilar changes in related cortical activity over time. It is noteworthy that this pilot study has demonstrated that the brain efficiency hypothesis maybe applicable in one process but not another. However, obviously, the generalizability of the findings is subject to certain limitations. First, our results were based on small samples of a unique population, elite race walkers, one must interpret the results from this pilot study with great caution. And we should be careful if extending the results to other populations. Future studies should recruit more such subjects, and then compare the brain activation pattern of them with those of a matched control group. Second, during action observation tasks and motor imagery tasks, in order to control the possible movements of the participants, we fixed their limbs, and removed the data with obvious movements. Future studies should use physiological recorders to monitor individuals’ limb movements.

## Conclusion

The present results showed that activation levels of the pars triangularis of the IFG, DLPFC, PMC and SMC, and S1 in EG were significantly lower than in the CG during the motor execution and observation tasks, and M1 of EG was significantly lower than the region of CG in motor execution task. During the motor imagery task, activation intensities of the DLPFC, PMC and SMC, and M1 in the EG were significantly higher than in the CG. Additionally, the activation intensities of the pars triangularis and PMC and SMC decreased with the passage of time in the motor execution and imagery tasks, whereas during the action observation task, no significant differences in these regions were found. This indicates that the brain efficiency hypothesis is not applicable to the motor imagery task; its applicability is related to the internal processing that the tasks involved.

## Data Availability

The raw data supporting the conclusions of this manuscript will be made available by the authors, without undue reservation, to any qualified researcher.

## Author Contributions

QZ, LS, YY, and XB designed the work and wrote the manuscript. QZ, YC, and SS acquired the data. QZ, PZ, and SY analyzed the data.

## Conflict of Interest Statement

The authors declare that the research was conducted in the absence of any commercial or financial relationships that could be construed as a potential conflict of interest.
